# Time trends of non-alcoholic beverage consumption among adults in Germany, 1990–2011

**DOI:** 10.1186/s12937-020-00538-8

**Published:** 2020-04-08

**Authors:** Anja Schienkiewitz, Marjolein Haftenberger, Gert B. M. Mensink

**Affiliations:** Unit 27 Health Behaviour, Department of Epidemiology and Health Monitoring, Robert Koch Institute, General-Pape-Straße 62-66, 12101 Berlin, Germany

**Keywords:** National Health Interview and Examination Survey, Germany, Time trend, Non-alcoholic beverages, Adults, Consumption frequency

## Abstract

**Background:**

In Germany, information on trends in non-alcoholic beverage intake over the last decades is sparse. The aim of this analysis is to examine trends in non-alcoholic beverage consumption among adults living in Germany between 1990 and 2011 with special focus on gender, age and education level.

**Methods:**

We used self-reported food frequency questionnaire information from 25 to 69 year old participants of three German National Health Interview and Examination Surveys conducted in 1990–1992 (*n* = 7466), 1997–1999 (*n* = 5825) and 2008–2011 (*n* = 5375) and focused on consumption frequency of fruit juice, soft drinks, water, tea and coffee. Positive answers in the categories “almost daily”, “daily” and “daily and more” were categorized as frequent beverage intake. Proportion estimates and 95%-CI were weighted to better reflect the German population using SAS 9.4 survey procedures for complex sample designs.

**Results:**

Between 1990–1992 and 2008–2011, the proportion of men and women who reported to frequently drink juice, soft drinks, water or tea has increased from 21.9% (95%-CI: 20.8–23.0%) to 27.2% (25.6–29.0%), 10.0% (9.0–11.1%) to 18.7% (17.3–20.3%), 59.1% (56.8–61.4%) to 87.6% (86.2–88.9%), and 32.2% (30.3–34.2%) to 39.2% (36.9–41.5%), respectively. Frequent consumption of coffee decreased from 80.6% (79.1–82.0%) in 1990–1992 to 74.9% (73.3–76.5%) in 1997–1999 and increased to 81.2% (79.8–82.6%) in 2008–2011. Frequent consumption of juice increased over time among men with middle and high education (17.7% (15.7–19.8%) to 26.4% (23.4–29.6%) and 22.9% (20.2–25.8%) to 32.7% (29.4–36.2%), respectively), whereas a similar increase was only seen among women with low education (19.8% (17.1–22.9%) to 28.4% (22.9–34.7%). Frequent soft drink consumption increased among men in all age and education groups but among women only in the 25 to 34 year age group and in the low education group. Frequent water consumption increased about 20% or more among men and women, in all age and education groups.

**Conclusions:**

The results show changes in non-alcoholic beverage consumption over the past two decades in Germany. Exploring non-alcoholic beverage intake over time is important for the evaluation of consumption patterns with regard to guidelines and to design appropriate prevention measures.

## Background

Sufficient intake of beverages, especially water, is required for most body functions and essential for human life. The German Nutrition Society (DGE e.V.) recommends that adults drink about 1.5 l (L) every day, preferably water or non-alcoholic and non-caloric beverages [1]. Daily intake of sugar sweetened and alcoholic beverages are explicitly not recommended. In Germany, data from the German National Nutrition Survey (NVS) II, conducted in 2005–2006 among individuals aged 14 to 80 years indicated a mean daily non-alcoholic beverage intake of about 2.3 L/day. Nearly half of the non-alcoholic beverage intake in the population was water, without significant differences in intake between men and women. Coffee and black tea accounted for about one quarter, fruit juice for around 11% and soft drinks for 9% among men and 4% among women of the non-alcoholic beverage intake [2]. Similar results were observed in the recent National Health Interview and Examination Survey (DEGS, 2008–2011): For adults aged 30 years and older, water was the most frequently consumed non-alcoholic beverage, whereas juices and soft drinks were less common. Nevertheless, in all observed age groups, men consumed on average more sugar sweetened beverages (SSBs) than women [3]. Based on data from the Federal Statistical Office of Germany, mineral water and soft drinks are the most consumed cold non-alcoholic beverages followed by fruit juices. Between 1995 and 2015, the per capita consumption of mineral water increased from 98 L to 154 L and decreased to 148 L in 2017. Soft drink consumption increased from 92 L to 126 L in 2013 and then decreased to 116 L in 2017. The per capita consumption of fruit juices was around 40 to 42 L between 1995 and 2005 and decreased to 32 L by 2017. Over the same period, per capita consumption of alcohol decreased from 165 L to 131 L [4, 5].

A trend analysis on alcohol intake among German adults over two decades has been published recently [6], indicating that harmful alcohol consumption decreased among men and women from 1990 to 2011. However, information on trends in non-alcoholic beverage intake over this period of time is sparse. It is estimated that about a quarter of the daily energy intake of American adults resulted from non-alcoholic beverages [7], hence, the consumption of non-alcoholic energy containing beverages may also be substantial among adults in Germany. Systematic reviews and meta-analysis suggests that SSBs can be an important source of individual energy intake and the consumption of energy dense soft drinks has an effect on body weight and cardiovascular risk factors [8–10].

For Germany, changes in the frequency of daily non-alcoholic beverage intake over the last two decades are unknown. Therefore, the aim of this analysis is to examine non-alcoholic beverage consumption trends from individual beverage intake information between 1990 and 2011 among adults living in Germany with special focus on gender, age and education level.

## Methods

### Study design and population

Data from three cross-sectional German National Health Interview and Examination Surveys (GNHIESs) with information about health status, risk factors and health behavior of the German adult population were used [11]. In a multistage sampling procedure 120 to 180 sample-points (study locations) were randomly selected. In a second step, in every sample point an age- and sex-stratified random sample of the population was selected from population-registries. The first survey (East/West Health Survey 1990–1992,) was conducted as the final National Examination Survey within the German Cardiovascular Prevention Study in the former western part of Germany in 1990–1991 and analogous after reunification in former eastern Germany in 1991–1992 (in the following called EW, conducted between 1990 and 1992) [12]. The second survey, the German National Health Interview und Examination Survey 1998 (GNHIES98) was conducted between 1997 and 1999 and the third survey between 2008 and 2011 (DEGS). The data collection consisted of an interview with questions about diagnosed medical conditions and medication, self-administered questionnaires asking, among others, items about sociodemographic characteristics and life style factors and health examinations (e.g. anthropometry, blood pressure, blood samples) [13–15].

All participants were informed about study objectives and data protection handling. Verbal consent was witnessed and formally recorded in the first and second survey. Subjects in the third survey provided written informed consent prior to their participation. The surveys were conducted according to the Federal and State Commissioners for Data Protection guidelines. DEGS was approved by the local ethics committee at Charité- Universitätsmedizin Berlin in October 2008 (ethics approval application document number: EA2/047/08). The implementation of the EW and GNHIES98 conforms to the principles of the Helsinki Declaration.

Since the final National Examination Survey of the Cardiovascular Prevention Study was part of the EW and included a study population aged 25 to 69 years, the analyses are restricted to this age range. The overall response rates ranged from 70% in 1990–1992 to 61% in 1997–1999 [12, 16]. For DEGS the sample consists of 4193 first-time participants (42% response) and 3959 persons, who had already participated in GNHIES98 (62% response). Non-participants were asked to complete a short questionnaire including information on socio-demographic and health-related characteristics [14]. The comparison between responders and non-responders of DEGS and between the overall net sample and the resident population of Germany demonstrated a high degree of representativeness [17].

### Measurements

In all surveys, dietary data were collected using self-administered food frequency questionnaires; however, there were differences in the obtained data across the surveys in food groups, reference time, intake frequencies and information on portion sizes. To compare the information across surveys we could only use the frequency information of intake since portion sizes were not asked in GNHIES98. There were some differences in the assessment of beverage intake: in EW, beverage intake was assessed using the question “How often do you consume beverages?”. Participants reported their consumption of fruit and vegetable juices, soft drinks, mineral water, coffee, and tea. The frequency categories were “never”, “once a month or less”, “2-3 times a month”, “about once a week”, “several times a week”, and “(almost) daily”. In GNHIES98, data on frequency of beverage intake during the past 12 months were collected for fruit and vegetable juices, tap and mineral water, soft drinks (including cola, soda, tonic water, lemonade), coffee, and black tea using the categories “never”, “once a month or less”, “2–3 times a month”, “about once a week”, “several times a week”, “daily or almost daily”, and “several times a day”. A detailed description of the dietary assessment method in DEGS has been published previously [18]. In brief, the frequency of beverage intake during the last 4 weeks were assessed for fruit juices, vegetable juices, tap and mineral and flavored water, sugar-containing soft drinks (including cola, soda, tonic water, lemonade, iced tea, energy drinks), coffee, and black, green, herbal an fruit tea. The frequency categories were “never”, “once a month or less”, “2–3 times a month”, “1-2 times a week”, “3-4 times a week”, “5-6 times a week”, “once a day”, “twice a day”, “three times a day”, “4-5 times a day”, and “more than 5 times a day”. To examine trends over time from these surveys with different dietary collection methods we standardized the information of non-alcoholic beverages and summarized the beverages to the groups “fruit and vegetable juice”, “soft drinks”, “water”, and “coffee”. The trends for tea intake were limited to EW and DEGS because the questions about tea intake in the GNHIES98 were different. The reported frequency categories in all three surveys were recoded into monthly frequencies and then categorized as “none” (never), “occasional” intake (less than 4 times a month/once a week to several times/week), and “frequent” intake (almost daily or more).

Age was categorized in age groups (25–34 y, 35–44 y, 45–54 y, 55–69 y). The education level was defined in accordance with the International Standard Classification of Education (ISCED, version 1997) and categorized as low (9 or 10 years: lower secondary), medium (11–13 years: upper secondary), and high (14 or more years: higher education) [19].

### Analyses

From EW 1990–1992 to DEGS 2008–2011, a total of 18,666 adults were interviewed and examined (1990–1992: *n* = 7466 in, 1997–1999: *n* = 5825, 2008–2011: *n* = 5375). Participants with missing responses to all non-alcoholic beverage items (overall *n* = 210; 1990–1992 *n* = 1, 1997–1999 *n* = 141, and 2008–2011 *n* = 68) were excluded from the analysis. Men and women with missing information for one particular beverage item were included, resulting in slightly different n for single beverage items.

All statistical analyses were performed with survey procedures for complex samples taking into account the cluster design effect using SAS release 9.4 (SAS Institute, Cary, NC, USA). All statistical analyses were weighted using weighting factors which adjust for differences in demographic characteristics compared to the official German population according to sex, age, education, federal state of residence, and community type at the time of each survey (31 December 1991 for EW; 31 December 1997 for GNHIES98, 31 December 2010 for DEGS [17]). Additionally, the weighting factor for DEGS considers the re-participation probability of GNHIES98 participants. From 1990 on the age structure of the German population has changed considerably and therefore analysis between 1990–1992, 1997–1999 and 2008–2011 were standardized to the age structure of the population as of 31 December 2010.

For descriptive analyses, age-standardized frequencies and 95% confidence intervals (CI) were calculated for gender, age and education groups. Age-standardized trends over time were calculated with logistic regression models. Statistical hypothesis were tested with α = 0.05.

## Results

Table 1 summarizes the age-standardized frequencies of non-alcoholic beverage intake across the three surveys. From 1990–1992 to 2008–2011, the proportion of adults with a frequent intake of fruit juice, water and tea increased from 21.9% (95%-CI: 20.8–23.0%) to 27.2% (25.6–29.0%), 59.1% (56.8–61.4%) to 87.6% (86.2–88.9%), and from 32.2% (30.3–34.2%) to 39.2% (36.9–41.5%), respectively. The proportion of adults with a frequent soft drink intake markedly increased from 10.0% (9.0–11.1%) in 1990–1992 to 19.4% (17.8–21.0%) in 1997–1999 and was 18.7% (17.3–20.3%) in 2008–2011. In comparison, the proportion of adults with a frequent coffee intake decreased between 1990–1992 and 1997–1999 from 80.6% (79.1–82.0%) to 74.9% (73.3–76.5%) and subsequently increased to 81.2% (79.8–82.6%) in 2008–2011.

**Table 1:** Trends in age- and sex-standardized proportions of frequent, occasional and non-consumption of non-alcoholic beverages among adults in Germany (%, 95-CI) ^a^

Consumption frequency ^b^	EW^**c**^		GNHIES98^**c**^		DEGS^**c**^		absolute difference (%)	p trend ^**d**^
	1990–1992		1997–1999		2008–2011		1990–2011	
	%	95%-CI	%	95%-CI	%	95%-CI	%	95%-CI
**Fruit and vegetable juice**								
n	7425		5657		5287			
Frequent	21.9	20.8–23.0	23.0	21.7–24.3	27.2	25.6–29.0	+ 5.3	< 0.0001
Occasional -	67.7	66.5–68.8	58.9	57.3–60.5	57.0	55.5–58.5	−10.7	< 0.0001
None	10.5	9.6–11.4	18.1	16.8–19.6	15.8	14.6–17.1	+ 5.3	< 0.0001
**Soft drinks**								
n	7451		5680		5290			
Frequent	10.0	9.0–11.1	19.4	17.8–21.0	18.7	17.3–20.3	+ 8.7	< 0.0001
Occasional -	40.8	39.3–42.4	47.1	45.4–48.8	47.3	45.5–49.1	+ 6.5	< 0.0001
None	49.1	47.4–50.9	33.6	31.9–35.3	34.0	32.4–35.6	−15.1	< 0.0001
**Water**								
n	7462		5680		5290			
Frequent	59.1	56.8–61.4	76.1	74.3–77.7	87.6	86.2–88.9	+ 28.5	< 0.0001
Occasional -	33.9	32.3–35.6	19.5	18.2–20.8	10.5	9.3–11.8	−23.4	< 0.0001
None	7.0	6.0–8.0	4.5	3.8–5.3	1.9	1.5–2.4	−5.1	< 0.0001
**Coffee**								
n	7461		5678		5296			
Frequent	80.6	79.1–82.0	74.9	73.3–76.5	81.2	79.8–82.6	+ 0.6	< 0.0001
Occasional -	14.6	13.4–15.8	14.4	13.3–15.6	11.6	10.5–12.7	−3.0	0.0002
None	4.9	4.3–5.5	10.7	9.6–11.8	7.2	6.3–8.1	+ 2.3	< 0.0001
**Tea**^**e**^								
n	7440				5220			
Frequent	32.2	30.3–34.2			39.2	36.9–41.5	+ 7.0	< 0.0001
Occasional -	54.5	52.8–56.2			40.4	38.8–42.1	−14.1	< 0.0001
None	13.3	12.2–14.5			20.4	18.7–22.2	+ 7.1	< 0.0001

^a^All data are weighted to the German population as of 31.12.2010

^b^None (never); occasional intake (answering categories: less than 4 times a month to several times a week); frequent intake (almost daily or more)

^c^*EW* East/West Health Survey, *GNHIES98* German National Interview and Health Examination Survey 1998, *DEGS* German Health Interview and Examination Survey for Adults

^d^p for trend, age-standardized

^e^FFQ-Question in EW about tea, in GNHIES98 only about black tea (no information for herbal tea available), and in DEGS for black and green tea, herbal and fruit tea

The proportion of men with frequent fruit and vegetable juice consumption increased significantly from 19.4% (17.9–21.0%) in 1990–1992 to 26.9% (24.7–29.2%) in 2008–2011 (Table 2). This increase was statistically significant in the 35–44 and 55–69 year age groups. The proportion of men who frequently consume juice increased over time in the middle and high education groups from 17.7% (15.7–19.8%) to 26.4% (23.4–29.6%) and 22.9% (20.2–25.8%) to 32.7% (29.4–36.2%), respectively, but not in the low education group. Among women in total and in all age groups, there was no statistically significant increase in the proportion of frequent juice consumers over time. There was, however, a significant increase over time among women in the low education group (19.8% (17.1–22.9%) to 28.4% (22.9–34.7%).

**Table 2:** Trends in frequent intake of fruit and vegetable juice (%, 95-CI) by gender, age groups and education level ^a^ among adults in Germany ^b^

	EW ^**c**^		GNHIES98^**c**^		DEGS^**c**^		absolute difference (%)	p trend^**d**^
	1990–1992		1997–1999		2008–2011		1990–2011	
	%	95%-CI	%	95%-CI	%	95%-CI	%	95%-CI
**Men**	19.4	17.9–21.0	20.1	18.5–21.9	26.9	24.7–29.2	+ 7.5	< 0.0001
**Age groups (years)**								
25–34	23.0	19.8–26.6	23.8	20.3–27.7	30.0	24.8–35.8	+ 7.0	n.s.
35–44	16.6	13.8–19.9	20.2	16.9–24.0	31.2	26.0–36.9	+ 14.6	< 0.0001
45–54	19.0	16.2–22.0	17.7	14.5–21.5	21.6	18.2–25.5	+ 2.6	n.s.
55–69	19.6	17.0–22.6	19.8	17.1–23.0	26.2	22.6–30.0	+ 6.6	0.009
**Education**								
Low	19.2	15.5–23.6	19.6	14.5–26.0	13.6	9.6–18.8	−5.6	n.s.
Middle	17.7	15.7–19.8	17.8	15.7–20.1	26.4	23.4–29.6	+ 8.7	< 0.0001
High	22.9	20.2–25.8	24.5	21.5–27.9	32.7	29.4–36.2	+ 9.8	< 0.0001
**Women**	24.4	22.7–26.1	25.9	24.1–27.7	27.6	25.3–30.0	+ 3.2	n.s.
**Age groups (years)**								
25–34	28.3	25.1–31.7	32.4	28.7–36.2	28.8	23.6–34.6	+ 0.5	n.s.
35–44	24.2	21.2–27.4	25.2	21.7–29.0	28.3	23.9–33.0	+ 4.1	n.s.
45–54	22.4	19.6–25.5	24.1	20.5–28.2	27.0	23.3–30.9	+ 4.6	n.s.
55–69	23.7	20.8–26.9	23.7	20.7–27.0	26.9	23.5–30.6	+ 3.2	n.s.
**Education**								
Low	19.8	17.1–22.9	20.2	17.0–23.9	28.4	22.9–34.7	+ 8.6	0.02
Middle	25.0	23.0–27.2	26.7	24.2–29.3	27.0	24.3–29.9	+ 2.0	n.s.
High	32.4	28.5–36.6	32.4	28.3–36.9	28.9	24.9–33.2	−3.5	n.s.

^a^Information on education based on the International Standard Classification of Education (ISCED, version 1997) low (9 or 10 years: lower secondary), medium (11–13 years: upper secondary), and high (14 or more years: higher education)

^b^All data are weighted to the German population as of 31.12.2010

^c^*EW* East/West Health Survey, *GNHIES98* German National Interview and Health Examination Survey 1998, *DEGS* German Health Interview and Examination Survey for Adults

^d^p for trend, age-standardized

An increase in frequent soft drink consumption is seen among men between 1990–1992 and 1997–1999 and remained similar until 2008–2011 (Table 3). Among women, no change over time is observed. In 2008–2011, 25.2% (22.9–27.7%) of men were twice as likely as women to be frequent soft drink consumers (12.1%, 10.5–13.9%). The proportion of men who consume soft drinks frequently increased in all age and education groups between 1990–1992 and 1997–1999 and remained almost constant thereafter. For women, a similar trend is only observed in the age group 25 to 34 years. In 1997–1999 and 2008–2011, the proportion of men with frequent soft drink consumption was lower in the high education group than in the middle and low education groups. Between 1990–1992 and 2008–2011 the proportion of women with a frequent soft drink intake increased in the low education group and decreased significantly in the high education group.

**Table 3:** Trends in frequent intake of soft drinks (%, 95-CI) by gender, age groups and education level ^a^ among adults in Germany ^b^

	EW ^**c**^		GNHIES98 ^**c**^		DEGS ^**c**^		absolute difference (%)	p trend ^**d**^
	1990–1992		1997–1999		2008–2011		1990–2011	
	%	95%-CI	%	95%-CI	%	95%-CI	%	95%-CI
**Men**	8.5	7.3–9.8	25.0	22.8–27.3	25.2	22.9–27.7	+ 16.7	< 0.0001
**Age groups (years)**								
25–34	9.4	7.6–11.7	42.8	38.4–47.3	37.0	31.4–43.0	+ 27.6	< 0.0001
35–44	8.2	6.2–10.8	28.8	24.9–33.0	31.7	27.0–36.7	+ 23.5	< 0.0001
45–54	7.9	6.1–10.1	19.0	15.8–22.7	22.6	18.8–26.8	+ 14.7	< 0.0001
55–69	8.5	6.6–10.7	15.0	12.4–18.1	13.8	11.1–17.2	+ 5.3	0.0006
**Education**								
Low	9.0	6.3–12.6	31.6	24.8–39.3	31.7	24.4–39.9	+ 22.7	< 0.0001
Middle	9.0	7.7–10.6	27.7	24.8–30.7	29.2	26.0–32.6	+ 20.2	< 0.0001
High	7.1	5.3–9.4	16.8	14.4–19.5	14.8	12.4–17.6	+ 7.7	< 0.0001
**Women**	11.6	10.3–13.1	13.7	12.1–15.4	12.1	10.5–13.9	+ 0.5	n.s.
**Age groups (years)**								
25–34	14.1	11.6–16.9	23.7	19.8–28.1	18.8	14.3–24.3	+ 4.7	0.0005
35–44	11.6	9.4–14.3	13.6	10.8–16.9	11.7	8.9–15.3	+ 0.1	n.s.
45–54	11.9	9.5–14.9	11.5	8.8–14.9	13.1	10.4–16.4	+ 1.2	n.s.
55–69	9.7	7.6–12.2	9.1	7.1–11.7	7.3	5.3–9.9	−2.4	n.s.
**Education**								
Low	14.1	11.9–16.7	15.7	12.5–19.6	21.5	16.9–26.9	+ 7.4	0.02
Middle	11.0	9.4–12.8	13.4	11.4–15.6	12.1	10.1–14.5	+ 1.1	n.s.
High	8.2	6.5–10.3	11.3	8.6–14.7	3.7	2.5–5.5	−4.5	< 0.0001

^a^Information on education based on the International Standard Classification of Education (ISCED, version 1997) low (9 or 10 years: lower secondary), medium (11–13 years: upper secondary), and high (14 or more years: higher education)

^b^All data are weighted to the German population as of 31.12.2010

^c^*EW* East/West Health Survey, *GNHIES98* German National Interview and Health Examination Survey 1998, *DEGS* German Health Interview and Examination Survey for Adults

^d^p for trend, age-standardized

**Fig. 1:**
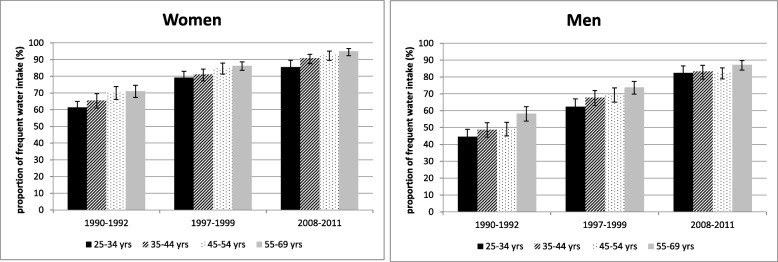
Trends in frequent intake of water (%, 95-CI) by gender and age group among adults in Germany

**Fig. 2:**
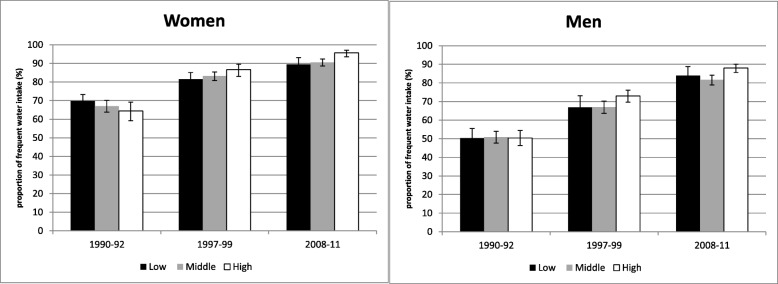
Trends in frequent intake of water (%, 95-CI) by gender and education (according to ISCED) among adults in Germany

The proportion with a frequent water intake increased steadily about 20% or more among men and women, in all age and education groups between 1990–1992 and 2008–2011 (Figs. 1 and 2). Overall, women are more likely to consume water frequently. Between 1997 and 1999 and 2008–2011, the proportion of men in the high education group with frequent water consumption is much higher than among men in the low education group. Among women, this gradient is only seen in 2008–2011.

## Discussion

Between 1990–1992 and 2008–2011, the proportion of adults with a frequent intake of juice, soft drinks and water as well as tea has increased in Germany. The increase in frequent soft drink intake was more pronounced between 1990–1992 and 1997–1999 compared to the later period. The proportion of consumers with a frequent coffee intake was lowest in the 1990s and increased between 1997–1999 and 2008–2011. Frequent consumption of juice increased among men in general and in the middle and higher education groups, whereas a similar change among women was only seen in the low education group. Between 1990–1992 and 1997–1999, frequent soft drink intake increased especially among men, in all age and education groups, and among women within the low education group. Furthermore, frequent water intake increased among men and women, and in all age and education groups.

Water, especially consumed as tap water, is easily accessible and fulfils the fluid needs of healthy individuals. Plain water contributes up to 80% of the total beverage intake in adults and is the main water source for all age groups [20]. The Federal Statistical Office of Germany presented an increase in the per capita consumption of mineral water by 50% from 1995 to 2015 [5]. In our analysis the proportion of adults who consume water frequently increased up to 30 percentage points. Recent changes in mineral water products, including different carbonation grades (no, medium, high), added flavor or fortification has broaden the possibilities to consume mineral water. In addition, the increased use of soda machines to carbonate water at home may have contributed to an increase of water consumption. Water is widely communicated as the first choice to quench thirst in the population and recommended in guidelines, so it may also be that people are more aware of this message [1].

In 2008–2011, one fifth of the German population reported a frequent intake of soft drinks. This is a similar dimension compared to data from the Behavioural Risk Factor Surveillance System (BRFSS), which indicates a prevalence of regular soda drinks ≥1 times daily by 17%, 21% among men and 14% among women [21]. For Germany, in 2005/2006 the mean daily consumption of soft drinks was on average 224 g among men and 88 g among women as reported by the NVS II and the oldest participants showed the lowest consumption of soft drinks [2]. This is in line with our observation on the prevalence of frequent intake: In 2008–2011, proportionally, twice as many men compared to women consumed soft drinks frequently and the highest frequent consumption was found in the youngest age group. This age dependent gradient is also described in a systematic assessment of individual-level beverage intake and country-level beverage availability data [22] and this gradient may contribute to the decrease in intake of total sugars as a percentage of energy over the lifespan [23]. Frequent soft drink intake in lower education compared to the higher education groups as seen in our data is consistent with findings from another survey which compared heavy consumption of SSBs among education and income groups [24].

The increase in the proportion of frequent soft drink intake from 1990–1992 to 1997–1999 could be due to the fact that various products of SSBs entered the market during this period. In the US, from 1977 to 2001 also larger portion sizes and increasing number of SSB servings were reported [7]. For Germany, official statistics indicate an increase until 2013 and a levelling off or a slight decrease in the per capita consumption of soft drinks thereafter [5]. Reports on the relation between carbohydrate intake and chronic disease like the one published by the German Nutrition Society in 2006 [25] may have increased health consciousness in relation to SSB consumption. Although reliable data are missing, increasing health consciousness is especially found among women and in the high educated groups [26], in which the prevalence of frequent soft drink intake in our observations dropped by 50%.

Fruit juice is an important source of vitamins and minerals, and usual intake of 100% fruit juice consumption is associated with better diet quality [27]. Trends in mean daily fruit juice consumption collected from three European dietary surveys and within NHANES indicate a decrease in fruit juice consumption among European countries (Spain, Italy, France) from 2007 to 2016 and in the US from 1999 to 2012 [28, 29]. For Germany the NVS II 2005/2006 showed a decrease in mean daily consumption of fruit juices with increasing age with the oldest participants having the lowest consumption of fruit juices and nectars [2]. However, the upward trend in the proportion of frequent juice intake seen in our data seems unusual, but not unexpected. The increase of adults who consume juice frequently was accompanied by the public health campaign “5 a day”, which started in Germany in the beginning of the 1990s. “5 a day” recommends eating five portions of fruit and vegetables every day, and it is possible to replace one portion by a smoothie or a glass of 100% fruit or vegetable juice [30]. Furthermore, the first smoothies were produced mid-2000 to 2010 and have become increasingly popular since then. Smoothies are made by raw fruit or vegetables with other added ingredients. They have a healthy image and were advertised to have a nutritional benefit and to improve the diet. In our observations frequent juice intake increased especially among men and middle and high education groups, but also among women in the low education groups. It could be that especially these groups changed their beverage consumption pattern from certain alcoholic beverages to non-alcoholic beverages: between 1995 to 2015 there was a decrease in per capita consumption of beer from 135.9 L to 105.9 L [5] and also national health survey data show a decline in harmful alcohol consumption among men and women between 1990–1992 and 2008–2011 [6]. Also, data from the longitudinal part of the German National Nutrition Monitoring (NEMONIT) indicate that men and women increased their non-alcoholic beverage consumption over a 6-year period between 2005 and 2013 [31]. We assume that the implementation of the “5 a day” public health policy as well as the easy access and availability of smoothies and the decrease in beer intake could have resulted in a compensation of alcoholic with non-alcoholic beverages with a healthy image. These changes could have resulted in an increased consumption frequency in fruit juice in 2008–2011 compared to the 1990s.

To our knowledge, this is the first detailed description of trends over two decades of frequent intake of juice, soft drinks and water among German adults using nationally representative data sources. However, some potential limitations of our results should be considered. A major limitation of comparing the three surveys is that the food frequency information is different in some aspects and a comparison of the three surveys could be based only on the intake frequency. Therefore, beverage intake could not be quantified in a comparable way (e.g. mean daily amounts). Furthermore, fruit juices have different contents in fruit, water and sugar, but this restriction was not specified in the earlier surveys. Therefore, the data did not allow for differentiation of fruit juice by percent fruit content. This limits the conclusions that can be drawn from the analyses. A more general limitation is that information of beverage consumption was based on self-reported consumption and respondents might not answer unbiased. In general, recall bias is a serious limitation in the collection of dietary intake data and under-reporting or selective reporting of consumption is common. Due to social desirability, less healthy beverage intake like of soft drinks could be underreported or healthy beverages such as water overestimated. Furthermore, although we used a survey specific weighting factor adjusting for differences in demographic characteristics from the official German population, it cannot be ruled out that the effects over time within the ISCED categories can be also explained by the different age distributions within these groups. Finally, cross-sectional studies are useful to evaluate frequency of beverage consumption and changes in beverage patterns [20].

## Conclusion

In conclusion, in contrast to the population wide reduction in alcoholic beverage consumption, the frequent intake of juice, soft drinks and water is increasing. An increase of water consumption is in line with the dietary recommendations. Nevertheless, a daily intake of fruit juice and soft drinks, which have high sugar contents, is explicitly not recommended by dietary guidelines. Exploring non-alcoholic beverage intake over time is important for the evaluation of consumption patterns with regard to guidelines and may help to design appropriate prevention measures.

## Data Availability

The data from EW, GNHIES98 and DEGS cannot be made publicly available. The informed consent from study participants did not cover public deposition of data and a publicly anonymized version of the analytical data set used in our current analysis would not comply with current data protection regulations in Germany as anonymized information could still be used in combination and/or with other data to identify DEGS study participants. However, the minimal data set underlying the findings presented in this article is archived in the ‘Health Monitoring’ Research Data Centre at the Robert Koch Institute (RKI) and can be accessed by all interested researchers on site. The ‘Health Monitoring’ Research Data Centre is accredited by the German Data Forum according to uniform and transparent standards. On-site access to the minimal data set is possible at the Secure Data Center of the RKI’s ‘Health Monitoring’ Research Data Centre (e-mail: fdz@rki.de).
